# Brain Tumor MRI Classification Using a Novel Deep Residual and Regional CNN

**DOI:** 10.3390/biomedicines12071395

**Published:** 2024-06-23

**Authors:** Mirza Mumtaz Zahoor, Saddam Hussain Khan, Tahani Jaser Alahmadi, Tariq Alsahfi, Alanoud S. Al Mazroa, Hesham A. Sakr, Saeed Alqahtani, Abdullah Albanyan, Bader Khalid Alshemaimri

**Affiliations:** 1 Faculty of Computer Sciences, Ibadat International University, Islamabad 44000, Pakistan; mumtazzahoor5@gmail.com; 2 Department of Computer System Engineering, University of Engineering and Applied Science (UEAS), Swat 19060, Pakistan; saddamhkhan@ueas.edu.pk; 3 Department of Information Systems, College of Computer and Information Sciences, Princess Nourah bint Abdulrahman University, P.O. Box 84428, Riyadh 11671, Saudi Arabia; asalmazroa@pnu.edu.sa; 4 Department of Information Systems and Technology, College of Computer Science and Engineering, University of Jeddah, Jeddah 21959, Saudi Arabia; tmalsahfi@uj.edu.sa; 5 Nile Higher Institute for Engineering and Technology, Mansoura 35511, Dakahlia, Egypt; heshamsakr535@nilehi.edu.eg; 6 Radiological Sciences Department, College of Applied Medical Sciences, Najran University, Najran 61441, Saudi Arabia; salqahtani@nu.edu.sa; 7 College of Computer Engineering and Sciences, Prince Sattam bin Abdulaziz University, Al-Kharj 16278, Saudi Arabia; a.albanyan@psau.edu.sa; 8 Software Engineering Department, King Saud University, Riyadh 11671, Saudi Arabia; balshemaimri@ksu.edu.sa

**Keywords:** brain tumor classification, deep learning, convolutional neural networks, magnetic resonance imaging

## Abstract

Brain tumor classification is essential for clinical diagnosis and treatment planning. Deep learning models have shown great promise in this task, but they are often challenged by the complex and diverse nature of brain tumors. To address this challenge, we propose a novel deep residual and region-based convolutional neural network (CNN) architecture, called Res-BRNet, for brain tumor classification using magnetic resonance imaging (MRI) scans. Res-BRNet employs a systematic combination of regional and boundary-based operations within modified spatial and residual blocks. The spatial blocks extract homogeneity, heterogeneity, and boundary-related features of brain tumors, while the residual blocks significantly capture local and global texture variations. We evaluated the performance of Res-BRNet on a challenging dataset collected from Kaggle repositories, Br35H, and figshare, containing various tumor categories, including meningioma, glioma, pituitary, and healthy images. Res-BRNet outperformed standard CNN models, achieving excellent accuracy (98.22%), sensitivity (0.9811), F1-score (0.9841), and precision (0.9822). Our results suggest that Res-BRNet is a promising tool for brain tumor classification, with the potential to improve the accuracy and efficiency of clinical diagnosis and treatment planning.

## 1. Introduction

The human brain is the most complicated and imperative organ in the body, governing the neurological system. The most deadly brain tumor is caused by erratic and out-of-control cell growth in the brain [1]. Patient survival depends on the type of glioma type; low-grade gliomas have survival rates of 5 years as high as 80%, whereas the survival rates of 5 years are under 5% for high-grade gliomas [2]. Timely brain tumor recognition and categorization is an imperative research topic in the clinical imaging domain, and it assists in choosing the most suitable treatment plan for a patient’s lifesaving.

Several screening methods, either invasive or non-invasive, are employed to identify tumors in the human brain [3]. Magnetic resonance imaging (MRI) is a preferable, less harmful scanning modality since it provides rich information about the location of brain tumors, shape, and size in medical images (MI), and is generally considered quicker, cheaper, and safer [4]. The manual assessment of brain MRIs is challenging for radiologists to identify and categorize brain tumors from MIs. A computer-aided diagnosis (CADx) is required to reduce the burden and assist radiologists or doctors with an MI assessment.

Many research areas are being explored in medical image analysis. It includes medical imaging domains like identification, detection, and segmentation [5,6,7,8,9,10,11]. Traditional ML approaches comprise numerous steps, pre-processing, feature extraction and selection, and classification. More discriminative feature acquisition is essential, as classification accuracy relies on obtained features [12,13].

In conclusion, conventional ML techniques have two key challenges in the feature extraction step: one, that it solely emphasizes low- or high-level attributes. Secondly, standard ML techniques rely on hand-crafted features that require significant prior knowledge, such as the position of the tumor in a medical scan. However, there is a considerable risk of human error. Designing an effective system to incorporate high- and low-level features with no human intervention is crucial. As brain tumor datasets are being expanded, there is a need for technological improvements in feature extraction focusing on confined and imbalanced MR imaging datasets of brain abnormalities and other irregularities of the human organs [14,15,16]. 

In recent times, deep learning (DL) methods have frequently been employed for brain MRI categorization, including patients with disabilities [17]. While feature mining and classification were integrated into self-learning, deep learning methods do not necessitate a manual process for feature extraction. The DL approach requires a dataset, and minimal pre-processing is required for selecting salient features in a self-learning way [18]. MR imaging categorization faces a significant challenge in diminishing the semantic space among high-level spatial details observed by a human assessor and low level acquired using the imagery mechanism. One of the well-known neural network models, convolutional neural networks (CNNs), specially designed for images, is utilized for feature extraction to acquire the important characteristics to categorize and minimize the semantic gap [19].

Recently, in many studies, CNNs have been widely employed to classify brain MRIs, and are validated on a different dataset of brain tumors [20,21,22,23,24,25,26]. A deep CNN-based model was proposed in [27] for brain MRI image categorization into distinct classes. The authors used brain MRI images from a publicly available dataset to prevent model ambiguity. The suggested model has a classification accuracy of 91.4%. Deepak and Ameer [28] employed a pre-train deep CNN, GoogLeNet, to extract key attributes using brain MR images and classify tumors into three classes with 98% accuracy. Ahmet and Muhammad [29] categorized brain MR images using various CNN models and attained satisfactory accuracy. They modify a pre-trained ResNet-50 DCNN by excluding the final five layers and introducing an additional eight layers. The model achieved the highest among all pre-trained models, with an accuracy of 97.2%. Sultan et al. [30] suggested a CNN-based deep learning model utilizing two publicly accessible datasets which have 3064 (glioma, meningioma, and pituitary tumors) and 516 (Grade II, Grade III, and Grade IV) brain medical scans. The proposed method has the best accuracy of 96.13% and 98.7%. Khwaldeh et al. [31] used several CNNs to classify brain MRI images and achieved good results. Using a modified pre-trained Alexnet CNN, they achieved a higher accuracy of 97.2%. Khan, M.A. et al. [32] developed a multi-model-based technique to differentiate brain tumors with DL. The presented system includes many stages. They used partial least-squares (PLS) to concatenate the features and ELM for classification. Their methodology stated improvements of 97.8%, 96.9%, and 92.5% on BraTs-2015, BraTs-2017, and BraTs-2018, respectively. Özyurt et al. [33] presented a technique for detecting brain tumors. They began with MRI tumor image segmentation with the NS-EMFSE algorithm. They obtained features from the segmented image using AlexNet, and then using the SVM, they detected and classified brain tumor images as benign or malignant with a 95.62% accuracy. 

However, most of these models are assessed on small-scale datasets due to the inaccessibility of the data repositories. Likewise, the majority of earlier research was based on pre-trained CNN models, which were developed generally for a dataset of natural images. Pretrained models are customized for the brain tumor task without designing them to distinguish brain tumor patterns; thus, it limits the use of pre-trained CNN models for brain tumor diagnosis.

In this study, a new deep CNN-based brain tumor classification scheme is developed for MRI image categorization. A novel CNN architecture, Res-BRNet, is suggested for brain tumor classification. Performance assessment is performed using standard measures like sensitivity, precision, F1-score, accuracy, and AUC-PR/ROC. Moreover, we have gathered a large dataset by collecting the brain MRI images of three tumor types and normal brain images from publicly accessible sources. The prediction ability of the developed approach is assessed on the test dataset and assessed with a comparison of numerous existing DCNNs, and the proposed technique concept is also compared with standard existing approaches. The proposed work has the following contributions:

- A novel deep residual and regional CNN architecture, Res-BRNet, has been developed for brain tumors classification.
- The proposed Res-BRNet integrates spatial and residual blocks to learn complex tumor patterns from brain MRIs, and it enhances the performance of the developed model for brain tumor classification.
- The developed Res-BRNet employed regional and boundary-based operations in a systematic order within the customized spatial and residual blocks to exploit spatial correlation information and textural variations from brain tumor MRIs.
- The systematic integration of residual and spatial blocks within the proposed Res-BRNet CNN improves the discriminative capability and generalization. Moreover, spatial blocks extract homogeneity and boundary-defined features at the abstract level. Furthermore, residual blocks at the target level effectively learn local and global texture variations of different brain tumors.

The rest of the manuscript is organized as follows: Section 2: incorporates the proposed methodology. The results and discussion are described in Section 3, and Section 4 concludes the entire paper.

## 2. Materials and Methods

**Figure 1:**
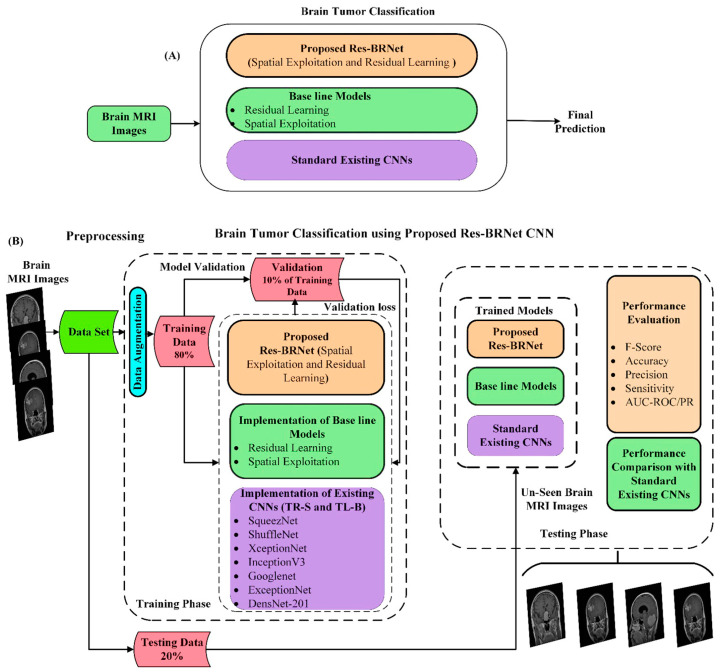
(**A**) Brief design of proposed brain tumor MRI image classification technique. (**B**) The detailed design of the proposed brain tumor MRI image classification technique.

In this work, a new deep residual and regional CNN architecture is designed for automated brain tumor classification from MRI images. The discriminating ability of the proposed classification method is empirically assessed using several standard performance measures, and the results are evaluated by comparing them with existing DCNNs. A better generalization is achieved by augmenting the training samples in the experimental setup. A brief and detailed description of the developed brain tumor classification technique is shown in Figure 1A,B.

### 2.1. Dataset

**Figure 2:**
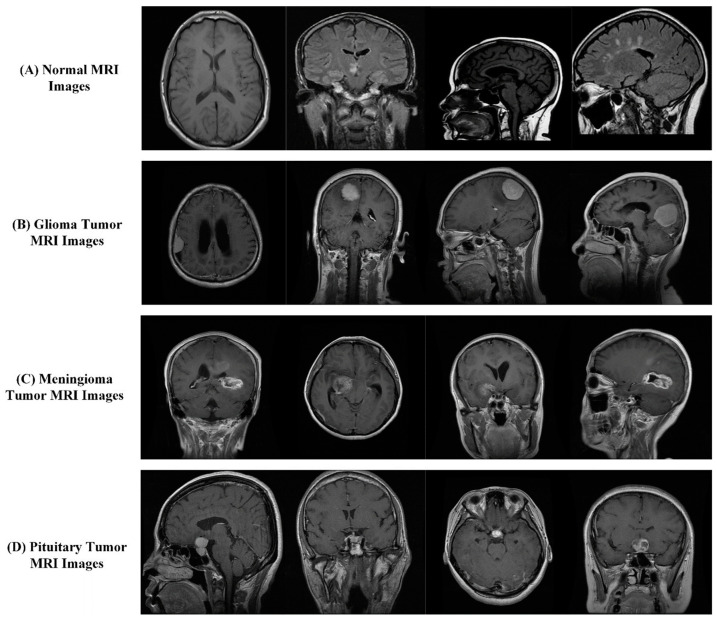
Example MRI images of normal and different types of tumors.

In this work, we have collected a dataset containing the 2D MRI images of healthy individuals and three diverse types of brain tumors. MRI scans of four classes are gathered from open-source Kaggle repositories [34], Br35H [35], and figshare [36], collected from 2005 to 2010 by Nanfang Hospital, Guangzhou, and General Hospital, Tianjin Medical University, China. For this experimental setup, we collected 2044 brain normal, 2352 glioma_tumor, 1645 meningioma_tumor, and 1831 pituitary_tumor MRI images from these repositories; hence, in nature, the acquired dataset is unbalanced, as shown in Table 1. Each image was resized according to the input size of DCNNs. Some of the four classes’ images are displayed in Figure 2.

### 2.2. Data Augmentation

On a small volume of data, deep learning models are generally overfitting. Thus, a significant amount of data are usually required to train deep CNNs and provide better generalizability. Data augmentation is generally employed for increasing the original dataset’s samples [5,37,38]. In this experiment, random rotation (02013360 degrees), scaling (0.5–1), sharing (± 0.05), and image reflecting (±1 range) are used to augment the dataset. These augmentation techniques are used to strengthen the model’s generalization.

### 2.3. Performance Metrics

The efficiency of the developed model was assessed using several standard evaluation measures. These measures include precision [39], sensitivity [40], accuracy [41], F1-score [42], PR, and ROC curves [43]. TP is defined as truly positive predictions, TN as truly negative predictions, FP as incorrectly positive predictions, and FN for incorrectly negative predictions. In Equation (1), accuracy is defined, calculating the total number of accurate selections. Accordingly, Sensitivity is in Equation (2), precision is denoted in Equation (3), and the F1-score is defined in Equation (4). In Equation (5), the standard error (S.E.) for the F1-score is calculated at a 95% confidence interval (CI), where z = 1.96 [44]. The CI is used as a statistical test to evaluate the uncertainty of the classification CNNs.

(1) $$Accuracy=\left(\frac{TP+TN}{TN+TP+FN+FP}\right)\times 100$$

(2) $$Sensitivity=\frac{TP}{TP+FN}$$

(3) $$Precision=\frac{TN}{TN+FP}$$

(4) $$F1-Score=\frac{2\times \left(P\times R\right)}{P+R}$$

(5) $$CI=z\sqrt{\frac{error(1-error)}{total\;instances}}$$

### 2.4. The Developed Deep Res-BRNet-Based Categorization

In this work, we exploit the learning capability of a deep CNN to acquire the tumor’s distinctive patterns in brain MRI scans. The strong potential of a deep CNN for learning specific features and patterns from images inspires us to employ them for classification and recognition tasks. Because of their effective learning capability, CNNs are largely employed for feature extraction and classification. In this proposed work, we designed a novel residual and regional CNN architecture using boundary- and region-based operations to classify tumor-specific abnormalities in brain MRI images and named it Res-BRNet. The proposed model is trained in an end-to-end way to learn the tumor-related patterns from MRI scans. The last fully connected layers, followed by softmax-based operation of the proposed deep CNNs, are used for the final classifications. The details of Res-BRNet are depicted in the section given below.

### 2.5. Structural Details of the Developed Res-BRNet

The architecture-level details of the developed Res-BRNet are inspired by standard image processing techniques [45,46]. It is developed to explore the hidden insights in MRI images. In this context, the region- and boundary-based operators with convolution operators are optimized in the proposed architecture to attain the brain tumor patterns excellently. In this work, we exploited spatial and residual blocks [47,48,49] as baselines to justify the advantages of region uniformity and boundary-related features for obtaining the tumor patterns using CNNs. 

**Figure 3:**
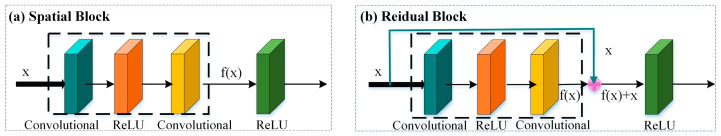
The difference in the process of (**a**) plain and (**b**) residual blocks.

As illustrated in Figure 3 in a spatial block, input x is fed into the operation block, and all operators are applied sequentially on input, and at the output operation block, we get T (x) = $f_{conv.}$(x), as sown in Equation (6). The difference between a feed-forward spatial block and residual learning is that residual blocks skip connections from input x to the output of the encoding block and add up with the output of the encoding block $f_{conv.}$(x). At the output of the residual block, we get T (x) = $f_{conv.}$(x) +x, as shown in Equations (7) and (8). As compared to the spatial block, residual learning facilitates the model to capture minor textural and contrast variations and also facilitates overcoming the vanishing gradient problem, as well as improves the learned feature maps and model’s convergence.

(6) $$T\left(x\right)=f_{conv.}\left(x\right)$$

(7) $$T\left(x\right)=f_{conv.}\left(x\right)+x$$

(8) $$f_{conv.}\left(x\right)=T\left(x\right)-x$$

The proposed Res-BRNet comprises three spatial blocks at the start, and four residual blocks are used after them. Every spatial block contains a single convolution layer (Equation (8)), batch normalization, and ReLU. The convolution layer exploits tumor-relating patterns, while ReLU performs as an activation function. To learn the region homogeneousness and boundary-related attributes of brain tumors, a max- or average-pooling operation is applied at the end of each spatial block, as shown in Equations (10) and (11). 

**Figure 4:**
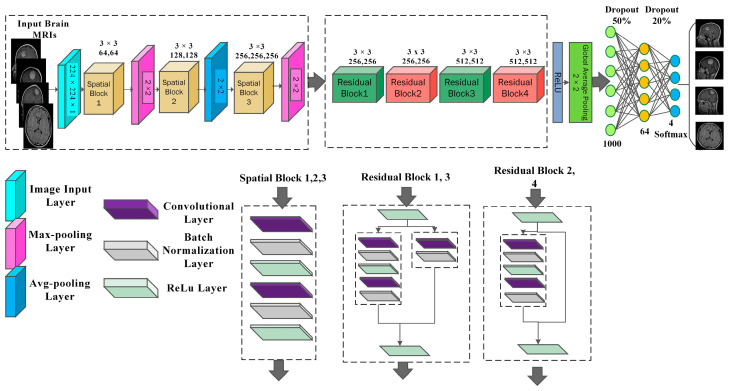
Blockwise details of the proposed Res-BRNet.

Figure 4 illustrates the architecture of the developed Res-BRNet. The fully connected (FC) layers stated in Equation (12) are applied in the designed architecture to attain particular features for classification. Dropout layers are used with FC layers to minimize the risk of overfitting.

(9) $$Z_{m,n}=\sum_{u=1}^{r}\sum_{v=1}^{s}Z_{m+u-1,\;n+v-1}\;k_{a,b}$$

(10) $$Z_{m,n}^{Avg}=\frac{1}{T^{2}}\sum_{u=1}^{t}\sum_{v=1}^{t}Z_{m+u-1,\;n+v-1}$$

(11) $$Z_{m,n}^{Max}={Max}_{u=1\ldots t,v=1\ldots t}Z_{m+u-1,\;n+v-1}$$

(12) $$Q=\sum_{b}^{B}\sum_{c}^{C}W_{d}Z_{c}$$

Z illustrates the source feature map of size × N, and the filter with size r×s is defined by k in the convolutional operator used in Equation (9). The output map of features is shown by Z. m and n have begun from 1 to (M−r+1) and (N−s+1), accordingly. As shown in Equations (9)–(11), we regulate the Zavg and Zmax methods, denoted by Z Avg and Z Max, similarly. In Equations (10) and (11), t indicates the average and max window dimensions. In Equation (12), the dense layer outcome is stated by Q, which employs global operation on $Z_{c}$. The FC-layer neurons are presented by $W_{d}$ and preserve essential features for the analysis. We used a kernel size of 3 × 3, padding of 0, 0, and ReLU as the activation function for Res-BRNet. Table 2 shows the used symbols and their description.

### 2.6. Benefits of the Proposed Res-BRNet for Image Content Analysis

Brain MRI scans reveal complex patterns with different intensity levels in distinct regions. Regional smoothness, textural differences, and edges make the basic structure of these patterns. In this study, the developed model is significantly improved by combining the convolutional operator (Equation (9)), enhancing the region homogeneity and boundary-based operations (Equations (10) and (11)), respectively, to differentiate the healthy instances from the tumor-affected MRI scans. In contrast to the developed model, the majority of existing CNN designs employ different convolutional combinations with simply one type of pooling layer to capture invariant features [47,48,50,51,52]. The proposed Res-BRNet employed regional and boundary-based operations in a systematic order within the modified spatial and residual blocks. The systematic use of boundary and regional operations within spatial blocks extract the brain tumor’s homogeneity and heterogeneity patterns, and boundary-related features. Additionally, the residual blocks significantly capture local and global texture variations of brain tumors. The following are the significance of applying the proposed idea in CNN:

- The developed residual and regional CNN architecture aimed to dynamically exploit image smoothness and sharpness, and it may efficiently optimize the level of smoothness and sharpening in harmony with the spatial features of an image.
- Implementing the spatial block with residual learning improves the overall detection ability of the model by acquiring textural features along with spatial correlation from MRI images.
- The systematic use of boundary and regional operations within spatial blocks helps enhance the region homogeneity of various regions. Using average pooling, the region operator helps smooth the regional variations and eliminates noise caused by distortions captured during MRI imaging. On the other hand, Res-BRNet is aided by boundary operators to acquire discriminative local features with the max pooling operation.
- Residual blocks aid the model in capturing textural and minor contrast variations and overcoming the vanishing gradient problem, which is generally produced in very deep architectures.
- Down-sampling is also performed during pooling operations, which increases the model’s robustness to small changes in the input image.

### 2.7. Employment of Existing CNNs

Competitive assessment is performed by implementing several existing deep CNN models, including SqueezeNet, ShuffleNet, VGG-16, Xception, ResNet-18, GoogleNet, Inception-V3, and DenseNet-201 [47,48,50,52,53,54,55,56,57]. Several researchers applied these CNNs to classify MRI images and they have been widely used for many image recognition tasks. Although these models’ block architecture and design changed, they all employed a single-pooling operation along the network or changed this for a stridden convolution operation to control complexity. To fine-tune these CNNs for brain tumor classification, we added FC and a classification layer, and employed them in an end-to-end manner.

### 2.8. Implementation Details

A brain MRI dataset was split into two sets, the 80% train set and 20% test set using the hold-out method. Furthermore, the train set was divided into train and validation data to select optimized parameters. ‘RMSprop’ [58] was employed for optimization with a ‘SquaredGradientDecayFactor’ of 0.95 throughout the training of CNNs. The learning rate was initially set to 0.0001 with the “LearnRateDropFactor” to be 0.4 and 40 epochs. A small-batch-based technique is used to train models on a batch size of 16 for every epoch. As an activation function, softmax was used, and cross-entropy loss has been reduced for all of the deep CNN optimizations. We have trained deep CNN models using hyper-parameters that have been experimentally optimized by holdout cross-validation techniques, including learning rate, batch size, and number of epochs. These models were executed on MATLAB-based simulations, utilizing a hardware setup consisting of a 2.90-GHz Dell Core i7-7500 CPU and a Nvidia^®^ GTX 1060 Tesla graphics card with CUDA support, Islamabad, Pakistan.

## 3. Results and Discussion

This study suggests a deep CNN-based system for identifying brain tumor patients using MRI images. We performed two different experiments for empirical evaluation of the developed technique. We initially explored the impacts of using simultaneously average and max pooling in spatial blocks of Res-BRNet. Secondly, a general assessment of brain tumor classification is carried out by comparing performances with well-known existing deep CNN models.

### 3.1. Efficiency Analysis of the Proposed Res-BRNet

**Figure 5:**
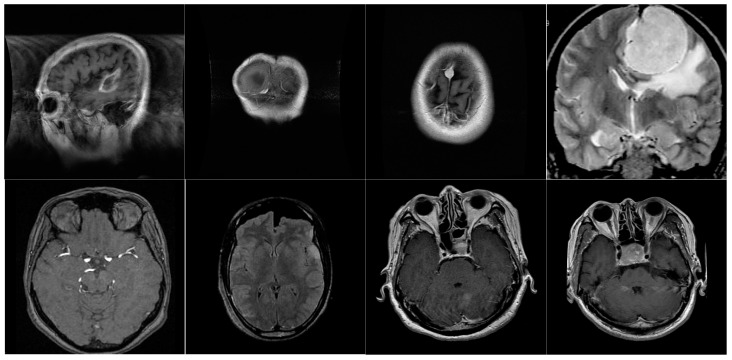
Normal and three tumor images misclassified by Res-BRNet.

**Figure 6:**
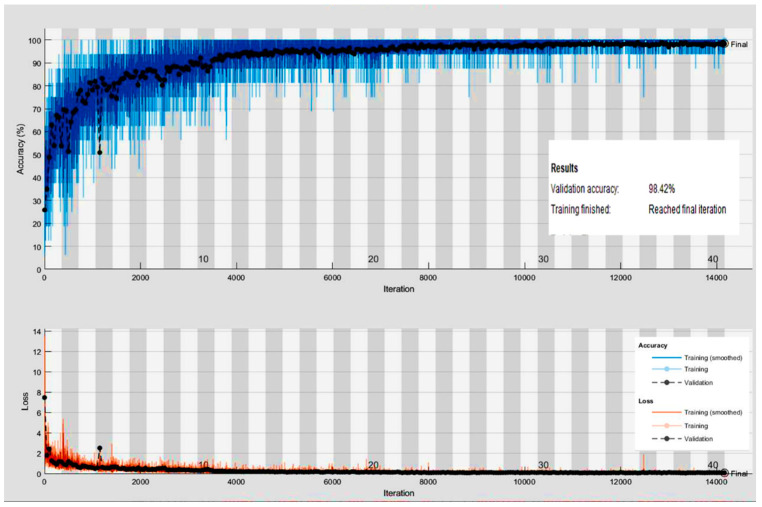
Plots of training the proposed Res-BRNet.

In a comprehensive experimental investigation, the proficiency of the developed Res-BRNet was assessed with well-known CNNs on unseen test data using the Accuracy, F1-score, Sensitivity, Precision, ROC, and PR-AUC. In contrast to accuracy, the F1-score tends to give more weight to precision and sensitivity. The proposed Res-BRNet model correctly classified 1553 samples of three brain tumors and normal instances. Likewise, the proposed Res-BRNet performs similarly by correctly identifying 463 gliomas, 321 meningiomas, 365 pituitary, and 404 normal individuals correspondingly. It is observed that a change in the region and boundary arrangements, as illustrated in Figure 3, improves the overall performance. Figure 5 displays some of the brain MRI images that Res-BRNet misclassifies. The input images have minimum intensity = 0, maximum intensity = 255, mean intensity = 128.5, and standard deviation of 64.3. The training loss and accuracy chart for Res-BRNet are presented in Figure 6. The developed CNN converges smoothly and quickly to achieve its optimal value, as seen in Figure 6. Low contrast, irregular sample patterns, and varying illumination variations are the probable reasons for misclassification. The generalization and robustness enhancement of test samples are achieved by using several data augmentation strategies while developing CNNs.

#### 3.1.1. Analysis of Performance with Baseline Methods

The significance of the anticipated idea is assessed by evaluating the performance, especially in comparison to residual learning and spatial exploitation-based architectures. Both baseline architectures, VGG-16 and ResNet-18, are almost as deep as Res-BRNet. Spatial block-based architectures exploit one type of down-sampling operation, and residual blocks use stridden convolution instead of pooling down.

Initially, both residual learning and spatial exploitation combinedly improve the classification ability of the proposed Res-BRNet, with an F1-score of 0.9385 and accuracy of 96.79%. Furthermore, employing both pooling operators (region and edge-based operators) in Res-BRNet improves the overall performance of the developed model, as shown in Table 3. Thus, according to performance comparison, the Res-BRNet consisting of region and edge-based operator shows exceptional performance compared to residual blocks and spatial block-based architectures in terms of F1-score (0.9641) and accuracy of (98.22%). The proposed Res-BRNet model significantly reduced the false positives and increased the true positives, leading to enhanced precision. 

#### 3.1.2. Analysis of Performance with Reported Techniques

The performance of the proposed work has also been compared to previously reported studies and exhibited in Table 4. Irmak [59] introduced two CNN models: (i) tumor detection (tumor or no tumor) and (ii) tumor type classification. The reported testing accuracy for tumor type classification was 92.66%. E. M. Senan et al. [60] performed different experiments for brain tumor analysis by combining deep learning and conventional ML methods and achieved the best performance, with 95.10% accuracy, 95.25% sensitivity, and 98.50% specificity with AlexNet + SVM. M. F. Alanazi et al. [61] developed a TL-based model to detect the subclass of the tumor. The developed transfer-learned model exhibited an accuracy of 95.75% for the brain MRI images. Another study by Kang et al. [62] utilized deep features to train an SVM model. However, the training–vector feature size was large and required a significant amount of computational time for training. 

**Figure 7:**
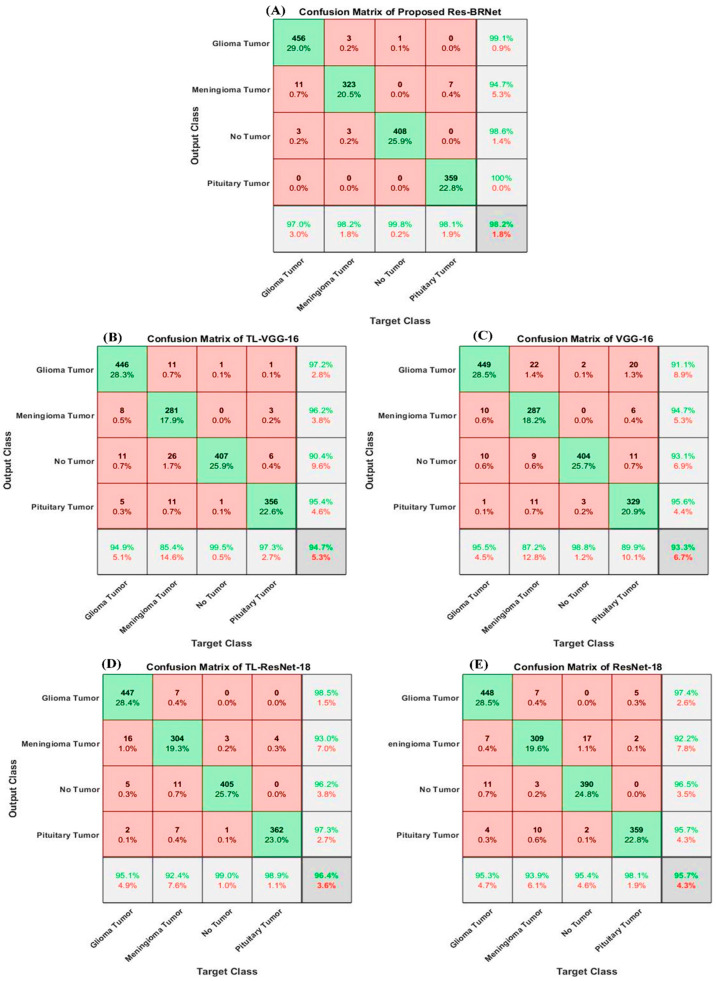
Confusion matrix-based performance assessment of the developed Res-BRNet and baseline architectures for different brain tumors.

**Figure 8:**
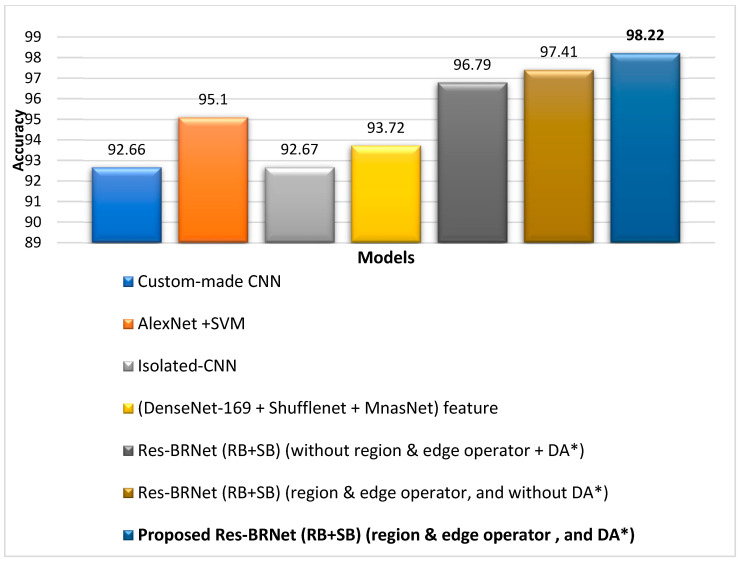
Performance assessment of the developed Res-BRNet with state-of-the-art CNN architectures. (* data augmentation (DA), spatial block (SB), residual block (RB)).

The proposed framework achieved a classification accuracy of 93.72% when tested on an unseen dataset. Table 4 and Figure 7 and Figure 8 show that the suggested Res-BRNet (with region and edge operator, and augmentation) considerably enhances the detection ability for all three brain tumors, as well as for normal MRI images compared to baseline residual learning and spatial exploitation architectures, without employing region and edge operators and data augmentation.

#### 3.1.3. Performance Assessment with Existing CNNs

The effectiveness of the developed Res-BRNet is compared with custom-made training-from-scratch and transfer learning-based (TL-based) existing CNNs, namely SqueezeNet, ShuffleNet, VGG-16, Xception, ResNet-18, GoogleNet, InceptionV3, and DenseNet-201. Table 5 and Table 6 illustrate that the proposed models’ performance analysis indicates that Res-BRNet is more efficient at identifying the patterns specific to the brain tumors in MRI scans with standard measures of accuracy and the F1-score. This performance of the proposed model improved by using average_ and max_pooling operations systematically in the designed CNN. Overall, the developed Res-BRNet achieved a performance gain in terms of accuracy (TR-SC to proposed Res-BRNet (1.2–11.06%)), (TL-B to proposed Res-BRNet (0.45–7.31)), as shown in Table 7. In general, the model is encouraged to learn highly discriminative features and fine-grained information from the raw MR image by the use of these opposing pooling operations.

#### 3.1.4. Feature Space-Based Performance Analysis 

**Figure 9:**
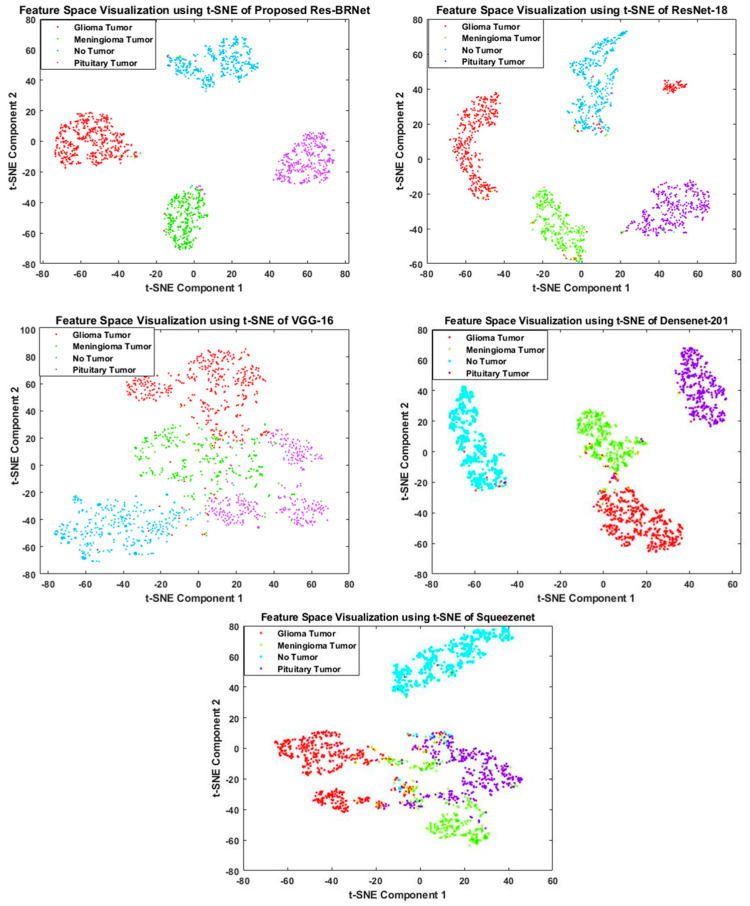
Feature space-based performance analysis of the developed Res-BRNet with baseline architectures (VGG-16, ResNet-18), best-performing TL-B model (DenseNet-201), and worst-performing TL-B model (SqueezeNet).

To understand decision-making behavior, the proposed Res-BRNet and best-performing baseline architectures, ResNet-18 and VGG-16, are evaluated to examine their learned feature spaces. Characteristics of the feature space responsible for the discrimination capability of a classifier. Features with classes distinguishably improve the model’s learning and lower the variance on distinct samples. T-distributed Stochastic Neighbor Embedding (t-SNE) [63] is an algorithm that is well-suited to visualize by embedding high-dimensional points in low dimensions based on similarities between points. Figure 9 illustrates the 2-D t-SNE plots for the proposed Res-BRNet, baseline models ResNet-18 and VGG-16, best-performing TL-B Densnet-201, and worst-performing TL-SqueezeNet using testing data. Data visualization shows that the feature space diversity is significantly improved by using both the boundary and regional operations, and it improves the model’s performance.

#### 3.1.5. ROC and PR-AUC-Based Analysis

**Figure 10:**
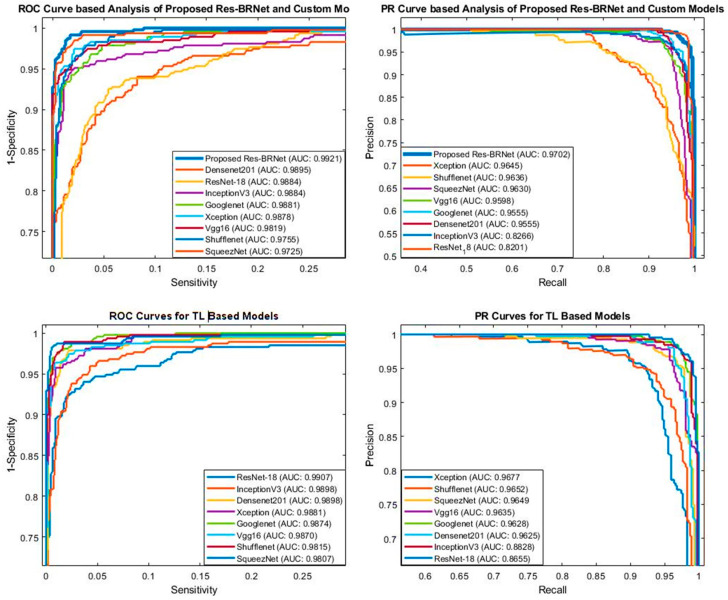
Detection rate analysis of the developed Res-BRNet with existing CNNs.

The ROC curve is essential to achieve the optimal analytic threshold for the classifier. ROC curve graphically displays the classifier distinction capability at possible threshold values. As shown in Figure 10, the proposed Res-BRNet achieved an AUC of (ROC_AUC of 0.9921 and PR_AUC of 0.9702). 

The brain MRI dataset includes patients with tumors. ROC and PR-AUC quantitative analysis proves that the suggested method enhances sensitivity by having the lowest false positive rate. This shows that the presented approach for classifying brain tumors has a lot of potential to be used in the analysis of brain tumors.

#### 3.1.6. Screening Effectiveness of the Proposed Technique

**Figure 11:**
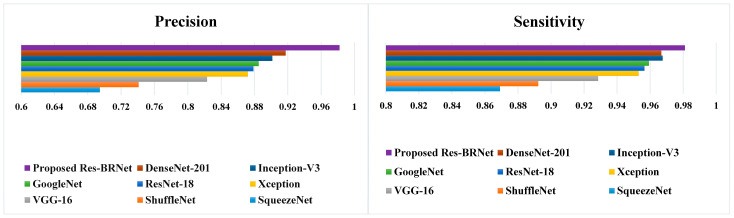
Performance analysis of the developed Res-BRNet with existing CNNs.

The precision and detection rates (sensitivity) are also critical metrics to evaluate a medical diagnostic system’s efficiency, where the costs of false positives and false negatives can be high. The brain tumor detection system needs to have a good detection performance. As seen in Figure 10 and Table 5 and Table 6, the detection rate and precision of the proposed approach are evaluated for brain MR images. As shown in the quantitative study (Figure 11), the Res-BRNet (Sensitivity: 0.9811, Precision: 0.9822) increases the prediction system’s accuracy and has a high prediction rate. Consequently, it is expected to help radiologists with good accuracy and may be utilized to enhance efficiency by decreasing the burden on medical professionals.

## 4. Conclusions

Brain tumor diagnosis at an early stage is crucial to cure the patient. Therefore, in this work, a new customized deep CNN model is developed to classify the brain MRI scans of meningioma, glioma, and pituitary tumor patients from healthy entities. The proposed model benefits from data augmentation and learning discriminative features using regional and boundary operators in the developed Res-BRNet. Moreover, the developed Res-BRNet employs spatial and residual ideas to acquire feature maps with diverse rich information, improving the capability to learn homogeneity, textural variation, and tumor’s structural patterns. The performance exploration of the developed is analyzed with the existing deep CNN models. The experiment results show that the proposed Res-BRNet outperforms existing CNN architectures, indicating accuracy and F1-score improvement. The developed method classifies brain tumors with an accuracy of 98.22%, an F1-Score of 0.9641, and sensitivity and precision of 0.9811 and 0.9822, respectively. The proposed approach will likely facilitate healthcare professionals in making diagnoses of brain tumors. Additionally, it motivates us to explore different forms of abnormalities in brain MRI and other medical images. Exploring novel techniques and methodologies to further optimize and validate the Res-BRNet model for clinical use may be considered in future.

## Figures and Tables

**Table 1:** Details of the collected dataset having MRI images of normal and different types of tumors.

	Glioma	Meningioma	Pituitary	Normal	Total
**Training (80%)**	1882	1316	1465	1635	6298
**Validation (10% of training)**	188	132	146	163	629
**Testing (20%)**	470	329	366	409	1574
**Total (100%)**	2352	1645	1831	2044	7872

**Table 2:** List of symbols used in this work.

Symbol	Description
$T\left(x\right)$	Output of spatial block
$T\left(x\right)$	Output of residual block
$f_{conv.}\left(x\right)$	Output of convolutional block
$Z_{m,n}$	Source feature map of size M×N
k	CNN’s filter with size r×s
Z	Output feature map
$Z_{m,n}^{Avg}$	Output of average pooling
$Z_{m,n}^{Max}$	Output of max pooling
Q	Output of dense layer

**Table 3:** Performance evaluation of the developed Res-BRNet with baseline architectures using the test data.

Model	Performance Comparison with Baseline Architecture CNNs			
	Accuracy %	Sensitivity	Precision	F1-Score
VGG-16 (SB + DA *)	93.32	0.9285	0.8231	0.8719
TL_VGG-16 (SB+ DA *)	94.66	0.9426	0.8569	0.8961
ResNet-18 (SB+ DA *)	95.67	0.9566	0.8788	0.9158
TL_ResNet-18 (RB+ DA *)	96.44	0.9641	0.8998	0.9303
Res-BRNet (RB+SB) (without region and edge operator + DA *)	**96.79**	**0.9661**	**0.9467**	**0.9385**
Res-BRNet (RB+SB) (region and edge operator, and without DA *)	**97.59**	**0.9712**	**0.9723**	**0.9446**
Proposed Res-BRNet (RB+SB) (region and edge operator, and DA *)	**98.22**	**0.9811**	**0.9822**	**0.9641**

Data augmentation (DA) *, spatial block (SB), residual block (RB).

**Table 4:** Performance evaluation of the developed Res-BRNet with reported techniques using the test data.

Model			Performance Comparison with Reported Techniques			
	Dataset	Class	Accuracy %	Sensitivity	Precision	F1-Score ± CI
Custom-made CNN [59]	Figshare	5	92.66	-	-	-
AlexNet + SVM [60]	MRI dataset	4	95.10	95.25	-	-
Isolated-CNN [61]	Kaggle	4	92.67	-	-	-
(DenseNet-169 + Shufflenet + MnasNet) feature [62]	BT-large-4c	4	93.72	-	-	-
**Proposed Res-BRNet**	**Figshare + Kaggle**	**4**	**98.22**	**0.9811**	**0.9822**	**0.9641 ± 0.0359**

**Table 5:** Performance analysis of the existing standard custom CNNs and the proposed Res-BRNet on the testing data with the confidence interval (CI).

Model	Performance Comparison with Custom-Made CNNs			
	Accuracy %	Sensitivity	Precision	F1-Score ± CI
SqueezeNet	87.16	0.8691	0.6946	0.7671 ± 0.2430
ShuffleNet	89.45	0.8923	0.7411	0.8047 ± 0.1953
VGG-16	93.32	0.9285	0.8231	0.8719 ± 0.1281
Xception	95.36	0.9531	0.8721	0.9101 ± 0.0899
ResNet-18	95.67	0.9566	0.8788	0.9158 ± 0.0842
GoogleNet	95.87	0.9593	0.8851	0.9196 ± 0.0804
Inception-V3	96.56	0.9676	0.9015	0.9331 ± 0.0669
DenseNet-201	97.01	0.9668	0.9175	0.9406 ± 0.0594
**Proposed Res-BRNet (with region and edge operator and augmentation)**	**98.22**	**0.9811**	**0.9822**	0.9641 ± 0.0359

**Table 6:** Performance analysis of the existing standard TL-based CNNs and the proposed Res-BRNet on the testing data with the confidence interval (CI).

Model	Performance Comparison with TL-Based CNNs			
	Accuracy %	Sensitivity	Precision	F1-Score ± CI
TL_SqueezeNet	90.91	0.9108	0.7685	0.8315 ± 0.1685
TL_ShuffleNet	92.31	0.9155	0.8056	0.8521 ± 0.1479
TL_VGG-16	94.66	0.9426	0.8569	0.8961 ± 0.1039
TL_Xception	96.37	0.9611	0.8996	0.9285 ± 0.0715
TL_ResNet-18	96.44	0.9641	0.8998	0.9303 ± 0.0697
TL_GoogleNet	96.37	0.9641	0.8985	0.9291 ± 0.0709
TL_Inception-V3	97.26	0.9711	0.9225	0.9459 ± 0.0541
TL_DenseNet-201	97.77	0.9778	0.9349	0.9557 ± 0.0443
**Proposed Res-BRNet (with region and edge operator, and augmentation)**	**98.22**	**0.9811**	**0.9822**	0.9641 ± 0.0359

**Table 7:** A performance gain of the developed Res-BRNet compared to TR-SC and TL-based models.

Improvement	Accuracy %	Sensitivity %	Precision %	F1-Score %
TR-SC to TL-B	0.76–3.75	1.1–4.45	1.74–7.39	1.51–6.44
TR-SC to Proposed Res-BRNet	1.2–11.06	1.43–11.02	6.47–28.76	2.35–19.07
TL-B to Proposed Res-BRNet	0.45–7.31	1.1–4.17	1.74–7.39	1.51–6.44

## Data Availability

The code of this paper is available at https://github.com/MumtazZahoor1/Res-BRNet-.git (accessed on 7 April 2024). The brain tumor datasets collected during the current study are available in the standard open access Kaggle [34], Br35H [35], and Figshare [36] repositories, and are verified by medical experts. Moreover, the datasets are available in publicly accessible repositories, which are described in Section 2.1. Correspondence and requests for materials should be addressed to S.H.K.

## References

[1] Behin A., Hoang-Xuan K., Carpentier A.F., Delattre J.-Y. (2003). Primary brain tumours in adults. Lancet.

[2] Miller K.D., Ostrom Q.T., Kruchko C., Patil N., Tihan T., Cioffi G., Fuchs H.E., Waite K.A., Jemal A., Siegel R.L. (2021). Brain and other central nervous system tumor statistics. CA. Cancer J. Clin..

[3] El-Dahshan E.-S.A., Mohsen H.M., Revett K., Salem A.-B.M. (2014). Computer-aided diagnosis of human brain tumor through MRI: A survey and a new algorithm. Expert Syst. Appl..

[4] Iftekharuddin K.M., Zheng J., Islam M.A., Ogg R.J. (2009). Fractal-based brain tumor detection in multimodal MRI. Appl. Math. Comput..

[5] Zahoor M.M., Qureshi S.A., Bibi S., Khan S.H., Khan A., Ghafoor U., Bhutta M.R. (2022). A New Deep Hybrid Boosted and Ensemble Learning-Based Brain Tumor Analysis Using MRI. Sensors.

[6] Khan A., Khan S.H., Saif M., Batool A., Sohail A., Khan M.W. (2022). A Survey of Deep Learning Techniques for the Analysis of COVID-19 and their usability for Detecting Omicron. arXiv.

[7] Khan S.H., Sohail A., Khan A., Lee Y.S. (2020). Classification and region analysis of COVID-19 infection using lung CT images and deep convolutional neural networks. arXiv.

[8] Asam M., Hussain S.J., Mohatram M., Khan S.H., Jamal T., Zafar A., Khan A., Ali M.U., Zahoora U. (2021). Detection of Exceptional Malware Variants Using Deep Boosted Feature Spaces and Machine Learning. Appl. Sci..

[9] Zahoor M.M., Qureshi S.A., Khan A., Rehman A.U., Rafique M. (2022). A novel dual-channel brain tumor detection system for MR images using dynamic and static features with conventional machine learning techniques. Waves Random Complex Media.

[10] Khan S.H., Sohail A., Khan A., Lee Y.-S. (2020). COVID-19 detection in chest X-ray images using a new channel boosted CNN. arXiv.

[11] Rauf Z., Sohail A., Khan S.H., Khan A., Gwak J., Maqbool M. (2023). Attention-guided multi-scale deep object detection framework for lymphocyte analysis in IHC histological images. Microscopy.

[12] Zahoora U., Khan A., Rajarajan M., Khan S.H., Asam M., Jamal T. (2022). Ransomware detection using deep learning based unsupervised feature extraction and a cost sensitive Pareto Ensemble classifier. Sci. Rep..

[13] Khan S.H., Iqbal J., Hassnain S.A., Owais M., Mostafa S.M., Hadjouni M., Mahmoud A. (2023). COVID-19 detection and analysis from lung CT images using novel channel boosted CNNs. Expert Syst. Appl..

[14] Alqahtani A., Zahoor M.M., Nasrullah R., Fareed A., Cheema A.A., Shahrose A., Irfan M., Alqhatani A., Alsulami A.A., Zaffar M. (2022). Computer Aided COVID-19 Diagnosis in Pandemic Era Using CNN in Chest X-ray Images. Life.

[15] Akkus Z., Galimzianova A., Hoogi A., Rubin D.L., Erickson B.J. (2017). Deep learning for brain MRI segmentation: State of the art and future directions. J. Digit. Imaging.

[16] Khan S.H., Khan A., Lee Y.S., Hassan M., Jeong W.K. (2022). Segmentation of shoulder muscle MRI using a new Region and Edge based Deep Auto-Encoder. Multimed. Tools Appl..

[17] Domingues I., Pereira G., Martins P., Duarte H., Santos J., Abreu P.H. (2020). Using deep learning techniques in medical imaging: A systematic review of applications on CT and PET. Artif. Intell. Rev..

[18] Goodfellow I., Bengio Y., Courville A. (2016). Deep Learning—Ian Goodfellow, Yoshua Bengio, Aaron Courville—Google Books.

[19] Asam M., Khan S.H., Akbar A., Bibi S., Jamal T., Khan A., Ghafoor U., Bhutta M.R. (2022). IoT malware detection architecture using a novel channel boosted and squeezed CNN. Sci. Rep..

[20] Gómez-Flores W., Pereira W.C.d.A. (2020). A comparative study of pre-trained convolutional neural networks for semantic segmentation of breast tumors in ultrasound. Comput. Biol. Med..

[21] Rawat W., Wang Z. (2017). Deep convolutional neural networks for image classification: A comprehensive review. Neural Comput..

[22] Arabahmadi M., Farahbakhsh R., Rezazadeh J. (2022). Deep Learning for Smart Healthcare—A Survey on Brain Tumor Detection from Medical Imaging. Sensors.

[23] Shirazi A.Z., Fornaciari E., McDonnell M.D., Yaghoobi M., Cevallos Y., Tello-Oquendo L., Inca D., Gomez G.A. (2020). The Application of Deep Convolutional Neural Networks to Brain Cancer Images: A Survey. J. Pers. Med..

[24] Xie Y., Zaccagna F., Rundo L., Testa C., Agati R., Lodi R., Manners D.N., Tonon C. (2022). Convolutional Neural Network Techniques for Brain Tumor Classification (from 2015 to 2022): Review, Challenges, and Future Perspectives. Diagnostics.

[25] Hoang Q.T., Yong K.-T., Liu X., Mahony D., Chaitarvornkit A., Cohen A., Grootswagers T. (2023). Detecting mild traumatic brain injury for athletes using SSVEP classification: A case study. Biomed. Signal Process. Control.

[26] Abdel-Nabi H., Ali M.Z., Awajan A. (2023). A multi-scale 3-stacked-layer coned U-net framework for tumor segmentation in whole slide images. Biomed. Signal Process. Control.

[27] Paul J.S., Plassard A.J., Landman B.A., Fabbri D. (2017). Deep learning for brain tumor classification. Med. Imaging 2017 Biomed. Appl. Mol. Struct. Funct. Imaging.

[28] Deepak S., Ameer P. (2019). Brain tumor classification using deep CNN features via transfer learning. Comput. Biol. Med..

[29] Çinar A., Yildirim M. (2020). Detection of tumors on brain MRI images using the hybrid convolutional neural network architecture. Med. Hypotheses.

[30] Sultan H.H., Salem N.M., Al-Atabany W. (2019). Multi-Classification of Brain Tumor Images Using Deep Neural Network. IEEE Access.

[31] Khawaldeh S., Pervaiz U., Rafiq A., Alkhawaldeh R.S. (2018). Noninvasive grading of glioma tumor using magnetic resonance imaging with convolutional neural networks. Appl. Sci..

[32] Khan M.A., Ashraf I., Alhaisoni M., Damaševičius R., Scherer R., Rehman A., Bukhari S.A.C. (2020). Multimodal Brain Tumor Classification Using Deep Learning and Robust Feature Selection: A Machine Learning Application for Radiologists. Diagnostics.

[33] Özyurt F., Sert E., Avci E., Dogantekin E. (2019). Brain tumor detection based on Convolutional Neural Network with neutrosophic expert maximum fuzzy sure entropy. Measurement.

[34] Br35H: Brain Tumor Detection 2020. Kaggle. https://www.kaggle.com/datasets/ahmedhamada0/brain-tumor-detection.

[35] Brain Tumor Classification (MRI). Kaggle. https://www.kaggle.com/datasets/sartajbhuvaji/brain-tumor-classification-mri.

[36] Jun C. (2017). Brain Tumor Dataset. https://figshare.com/articles/brain_tumor_dataset/1512427.

[37] Shorten C., Khoshgoftaar T.M. (2019). A survey on Image Data Augmentation for Deep Learning. J. Big Data.

[38] Khan A., Sohail A., Zahoora U., Qureshi A.S. (2020). A survey of the recent architectures of deep convolutional neural networks. Artif. Intell. Rev..

[39] Buckland M., Gey F. (1994). The relationship between recall and precision. J. Am. Soc. Inf. Sci..

[40] Davis J., Goadrich M. The relationship between Precision-Recall and ROC curves. Proceedings of the 23rd International Conference on Machine Learning, ACM.

[41] Diebold F.X., Mariano R.S. (2002). Comparing predictive accuracy. J. Bus. Econ. Stat..

[42] Sokolova M., Japkowicz N., Szpakowicz S. (2006). Beyond Accuracy, F-Score and ROC: A Family of Discriminant Measures for Performance Evaluation.

[43] Cortes C., Mohri M. (2005). Confidence intervals for the area under the ROC Curve. Advances in Neural Information Processing Systems 17.

[44] DiCiccio T.J., Efron B. (1996). Bootstrap confidence intervals. Stat. Sci..

[45] Hussain S., Khan A. (2020). Coronavirus Disease Analysis using Chest X-ray Images and a Novel Deep Convolutional Neural Network. Photodiagnosis Photodyn. Ther..

[46] Mallick A., Roy S., Chaudhuri S.S., Roy S. Optimization of Laplace of Gaussian (LoG) filter for enhanced edge detection: A new approach. Proceedings of the International Conference on Control, Instrumentation, Energy and Communication, CIEC 2014.

[47] He K., Zhang X., Ren S., Sun J. (2015). Deep Residual Learning for Image Recognition. Proc. IEEE Comput. Soc. Conf. Comput. Vis. Pattern Recognit..

[48] Simonyan K., Zisserman A. (2014). Very deep convolutional networks for large-scale image recognition. arXiv.

[49] Khan S.H., Shah N.S., Nuzhat R., Majid A., Alquhayz H., Khan A. (2022). Malaria parasite classification framework using a novel channel squeezed and boosted CNN. Reprod. Syst. Sex. Disord..

[50] Huang G., Liu Z., Van Der Maaten L., Weinberger K.Q. Densely connected convolutional networks. Proceedings of the 30th IEEE Conference on Computer Vision and Pattern Recognition, CVPR 2017.

[51] Zagoruyko S., Komodakis N. (2016). Wide Residual Networks. Proc. Br. Mach. Vis. Conf..

[52] Iandola F.N., Moskewicz M.W., Ashraf K., Han S., Dally W.J., Keutzer K. (2016). SqueezeNet. arXiv.

[53] Zhang X., Zhou X., Lin M., Sun J. ShuffleNet: An Extremely Efficient Convolutional Neural Network for Mobile Devices. Proceedings of the IEEE Computer Society Conference on Computer Vision and Pattern Recognition.

[54] Chollet F. Xception: Deep learning with depthwise separable convolutions. Proceedings of the 2017 IEEE Conference on Computer Vision and Pattern Recognition (CVPR).

[55] Szegedy C., Liu W., Jia Y., Sermanet P., Reed S., Anguelov D., Erhan D., Vanhoucke V., Rabinovich A., Liu W. Going deeper with convolutions. Proceedings of the 2015 IEEE Conference on Computer Vision and Pattern Recognition (CVPR).

[56] Yadav S.S., Jadhav S.M. (2019). Deep convolutional neural network based medical image classification for disease diagnosis. J. Big Data.

[57] Szegedy C., Vanhoucke V., Ioffe S., Shlens J., Wojna Z. Rethinking the Inception Architecture for Computer Vision. Proceedings of the IEEE Computer Society Conference on Computer Vision and Pattern Recognition.

[58] Ruder S. (2016). An Overview of Gradient Descent Optimization Algorithms. arXiv.

[59] Irmak E. (2021). Multi-Classification of Brain Tumor MRI Images Using Deep Convolutional Neural Network with Fully Optimized Framework. Iran. J. Sci. Technol. Trans. Electr. Eng..

[60] Senan E.M., Jadhav M.E., Rassem T.H., Aljaloud A.S., Mohammed B.A., Al-Mekhlafi Z.G. (2022). Early Diagnosis of Brain Tumour MRI Images Using Hybrid Techniques between Deep and Machine Learning. Comput. Math. Methods Med..

[61] Alanazi M.F., Ali M.U., Hussain S.J., Zafar A., Mohatram M., Irfan M., AlRuwaili R., Alruwaili M., Ali N.H., Albarrak A.M. (2022). Brain Tumor/Mass Classification Framework Using Magnetic-Resonance-Imaging-Based Isolated and Developed Transfer Deep-Learning Model. Sensors.

[62] Kang J., Ullah Z., Gwak J. (2021). Mri-based brain tumor classification using ensemble of deep features and machine learning classifiers. Sensors.

[63] Van Der Maaten L., Hinton G. (2008). Visualizing Data using t-SNE. J. Mach. Learn. Res..

